# Clinical and Epidemiologic Features of *Mycoplasma pneumoniae* Infection Among Adults Hospitalized with Community-acquired Pneumonia

**DOI:** 10.7150/ijms.99233

**Published:** 2024-11-11

**Authors:** Preeta K. Kutty, Seema Jain, Maureen H. Diaz, Wesley H. Self, Derek Williams, Yuwei Zhu, Carlos G. Grijalva, Kathryn M. Edwards, Richard G. Wunderink, Jonas Winchell, Lauri A. Hicks

**Affiliations:** 1 Centers for Disease Control and Prevention, Atlanta, Georgia, USA.; 2 California Department of Public Health, California, USA.; 3 Vanderbilt University School of Medicine, Nashville, Tennessee, USA.; 4 Northwestern University Feinberg School of Medicine, Chicago, Illinois, USA.

**Keywords:** *Mycoplasma pneumoniae*, adults

## Abstract

**Background/Purpose:** The burden and epidemiology of *Mycoplasma pneumoniae* (Mp) community-acquired pneumonia (CAP) among hospitalized U. S. adults (≥ 18 years) are poorly understood.

**Methods:** In the Etiology of Pneumonia in the Community (EPIC) study, we prospectively enrolled 2272 adults hospitalized with radiographically-confirmed pneumonia between January 2010—June 2012 and tested nasopharyngeal/oropharyngeal swabs for Mp by real-time polymerase chain reaction (PCR). Clinical and epidemiological features of Mp-PCR-positive and -negative adults were compared using logistic regression. Macrolide susceptibility was assessed by genotyping isolates.

**Results:** Among 2272 adults, 43 (1.8%) were Mp-PCR-positive (median age: 45 years); 52% were male, and 56% were non-Hispanic white. Only one patient had Mp macrolide resistance. Four (9%) were admitted to the intensive care unit (ICU). No in-hospital deaths were reported. Of the 9 (21%) who received an outpatient antibiotic ≤5 days pre-admission, 2 (22%) received an antibiotic with Mp activity. Variables significantly associated with higher odds of Mp detection included age {18-29 years [(adjusted odds ratio (aOR): 11.7 (95% confidence interval (CI): 5.1- 26.6) versus ≥50 years]} and radiographic lymphadenopathy [aOR: 3.5 (95% CI: 1.2- 9.3)].

**Conclusions:** *M. pneumoniae*, commonly known to cause “walking pneumonia”, was detected among hospitalized adults, with the highest prevalence among young adults. Although associated with clinically non-specific symptoms, approximately one out of every ten patients were admitted to the ICU. Increasing access to *M. pneumoniae* point-of-care testing could facilitate targeted treatment and avoid hospitalization.

## Introduction

*Mycoplasma pneumoniae* is a known cause of adult community-acquired pneumonia (CAP) 1-3. In addition to sporadic infection, *M. pneumoniae* can cause large community- and facility-based pneumonia outbreaks, especially in congregate settings such as universities, households, military installations, and healthcare facilities 1, 3-14. *M. pneumoniae* also causes upper respiratory infections and, in rare instances, extra-pulmonary manifestations (e.g., encephalitis and pericarditis), usually after respiratory disease 2, 15-20. Person-to-person aerosol spreads particularly close contact with and exposure to an infected person, which can result in the acquisition of the disease 2, 4, 21-25. Long incubation periods (6-32 days) and the persistence of the organism in the respiratory tract after symptom resolution contribute to prolonged transmission 2, 4, 26, 27.

The Centers for Disease Control and Prevention (CDC) Etiology of Pneumonia in the Community (EPIC) study was a prospective multi-center and population-based study of the etiologies of hospitalized CAP among adults in the United States conducted from 2010 to 2012 28. In this study, *M. pneumoniae* was the second most common bacterial pathogen detected after *Streptococcus pneumoniae* among hospitalized adults (2%), with an estimated incidence of 0.5 (95% confidence interval (CI): 0.4-0.7) cases per 10,000 adults per year. 28. Using this dataset, we describe the epidemiological and clinical characteristics of *M. pneumoniae* (Mp) among hospitalized adults with CAP in the EPIC study, comparing the characteristics of patients with and without *M. pneumoniae*.

## Methods

### Study population

The adult EPIC study details, published elsewhere, included five hospitals: three in Chicago, Illinois, and two in Nashville, Tennessee 28. Adults (≥18 years old) with clinical and radiographically confirmed pneumonia were enrolled from January 2010 through June 2012 from the five hospitals 28. Each site's and CDC's institutional review board approved the study protocol. Inclusion criteria were evidence of acute infection (reported fever or chills, documented fever or hypothermia, leukocytosis or leukopenia, or new altered mental status), evidence of acute respiratory illness (new cough or sputum production, chest pain, dyspnea, tachypnea, abnormal lung examination, or respiratory failure), and chest radiography findings consistent with pneumonia. Specific exclusion criteria included recent hospitalization, previous enrollment in the EPIC study, residence in an extended-care facility, an alternative diagnosis of a respiratory disorder or presence of a tracheostomy tube, cystic fibrosis, neutropenic cancer, recent solid-organ or hematopoietic stem-cell transplant, current graft-versus-host disease or bronchiolitis obliterans, or human immunodeficiency virus infection (with a CD4 cell count <200 per cubic millimeter). After informed consent was obtained, patients or their caregivers were interviewed, and medical charts were abstracted to collect clinical and epidemiological information. A dedicated study radiologist at each site reviewed all radiographic films to make a final determination of pneumonia. Radiographic evidence of pneumonia was defined as the presence of consolidation, other infiltrate, or pleural effusion 28. A convenience sample of asymptomatic adults from the Nashville study site (n=238) who presented for non-acute care to a general medicine clinic was enrolled weekly from November 1, 2011, to June 30, 2012 (*Supplementary material*) 28.

### Specimen collection and laboratory testing

Blood, urine, and respiratory specimens were obtained for bacterial and viral pathogen testing. 28. Nasopharyngeal/oropharyngeal (NP/OP) swabs were tested using CDC-developed real-time polymerase chain-reaction (PCR) assays. Mp-PCR-positive specimens were confirmed at CDC with multiplex PCR, culture, and molecular characterization, including macrolide susceptibility genotyping (*Supplementary material*) 29.

### Case definitions

An enrolled patient with an Mp PCR-positive specimen at the study site was considered to have Mp CAP (Mp-PCR-positive). If Mp was not detected by PCR but another pathogen was detected, the patient was considered to have CAP without Mp (Mp-PCR-negative). Bacterial and viral pneumonia, and pneumonia without a pathogen detected, are defined in the Supplementary material. Co-detection was defined as detecting ≥1 other bacterial or viral pathogens. Additional definitions are provided in the Supplementary material.

### Statistical analysis

We compared characteristics of adults with Mp-PCR-positive and -negative samples using Pearson chi-square or Fisher's exact tests for categorical variables and Mann-Whitney U and Wilcoxon signed-rank test for continuous variables, as appropriate. We assessed demographics, clinical features, and illness severity based on intensive care unit (ICU) admission, mechanical ventilation, death, and CURB-65 and Pneumonia Severity Index (PSI) scores 30, 31. Stratified analyses were conducted based on whether a pathogen (bacterial or viral) was detected or not.

We used multivariable logistic regression to compare Mp-PCR-positive and negative patients to explore characteristics independently associated with Mp detection. Variables with a P-value <0.20 on bivariate analysis and those with known biological or epidemiological plausibility were considered candidates for multivariable models. We fitted models using all candidate variables and automated stepwise procedures and then fitted alternate models using only selected variables. We used the Akaike information criterion (AIC) to help select among alternate models - this statistic simultaneously accounts for the goodness of fit and complexity of the tentative models. To resolve collinearity between the study hospital and race in the final model, we only controlled for the study hospital. All statistical tests were interpreted in a 2-tailed fashion to estimate p-values and 95% confidence intervals (CI) and used SAS version 9.3 (SAS Institute Inc., Cary, NC).

## Results

### Study population

From January 2010 to June 2012, 2488 (68%) of 3634 eligible adults were enrolled in the EPIC study; 2320 (93%) met the radiographic criteria of pneumonia. Of these 2320 adults, 2272 (98%) had Mp PCR testing performed onsite, and 43 (1.8%) were Mp-PCR-positive. A total of 810 (36%) were Mp-PCR-negative but had another pathogen detected, including rhinovirus (n=193, 24%), influenza A/B viruses (n=131, 16%), and *S. pneumoniae* (n=115, 14%) as the most detected pathogens.

### Bivariate and stratified analyses

Below, we present the findings of the comparison between adults who were Mp-PCR-positive in comparison with those who were Mp-PCR-negative and had another pathogen detected, hereby designated as Mp-PCR-negative. The Supplementary Material provides the demographic characteristics of adults hospitalized for Mp CAP (*Supplemental Table 1*) and the unadjusted analyses of select epidemiologic features among adults hospitalized for CAP with Mp and those without Mp, including those in whom no pathogen was detected (*Supplemental Table 2*).

#### Patient characteristics

Among the 43 Mp-PCR-positive patients, 52% were male, and 56% were non-Hispanic white (*Table 2*). In unadjusted analyses, Mp-PCR-positive patients were younger than Mp-PCR-negative (median 45 years vs. 57 years; P<0.01), had a longer duration of symptoms before hospitalization (median: 6.6 vs. 4.1 days; P<0.01), and were more likely to have a cough (100% vs. 91%; P=0.03), chest pain (63% vs. 43%, P=0.03), radiographic consolidation (79% vs. 64%, P = 0.04) or hilar lymphadenopathy (14% vs 6%, P=0.047). Mp-PCR-positive patients were less likely to have comorbidities (42% vs. 78%, P<0.01) or leukocytosis (28% vs. 49%, P <0.01) (*Table 1*). Overall, median hospital length of stay (LOS) was shorter (2 vs. 4 days, P<0.01) for Mp-PCR-positive versus Mp-PCR-negative patients.

#### Severity

No deaths were reported among Mp-PCR-positive patients during hospitalization, while 16 (2%) of Mp-PCR-negative patients died. Mp-PCR-positive patients were less likely to have ICU admission (9% (4) vs. 26% (210), P<0.01), and none underwent mechanical ventilation. The Mp-PCR-positive patients admitted to the ICU (n=4) had ≥1 co-morbidity. Among the Mp-PCR-negative patients admitted to the ICU, 62 (29%) required mechanical ventilation, and 15 (7%) died. For Mp-PCR-positive patients in the ICU, the median LOS was shorter (5 vs. 7.5 days, P=0.3) when compared with Mp-PCR-negative. No significant difference in CURB-65 scores between the groups was found, but Mp-PCR-positive patients were significantly more likely to be in lower PSI classes than Mp-PCR-negative (*Table 1*).

#### Antibiotic treatment

A higher proportion of Mp-PCR-positive patients received outpatient antibiotics within 5 days prior to admission compared with Mp-PCR-negative patients (21% vs. 11%; P=0.04) (*Table 1*); penicillins were more commonly administered among Mp-PCR-positive than Mp-PCR-negative (56% vs. 17%, P=0.01) patients. Of nine Mp-PCR-positive patients who received an outpatient antibiotic before admission, only two received an antibiotic with activity against Mp.

During hospitalization, all Mp-PCR-positive patients received an antibiotic with activity against Mp: 14 (33%) received a macrolide only, 14 (33%) received a macrolide and a fluoroquinolone, 8 (19%) received a fluoroquinolone only, 3 (7%) received a fluoroquinolone and a tetracycline, 2 (5%) received a macrolide and tetracycline, and 2 (5%) received a tetracycline only.

A single patient had a macrolide-resistant Mp strain, as determined by sensitivity testing at the CDC laboratory, without a history of macrolide receipt prior to admission (*Supplementary material*). Upon hospital admission, the patient received azithromycin (day 1) and ceftriaxone (day 2) and was discharged on levofloxacin.

#### Co-detections

The prevalence of co-detections between Mp-PCR-positive and Mp-PCR-negative patients was comparable (6 [14%] vs. 104 [13%], P=0.8). Among the six Mp-PCR-positive patients with a co-detected pathogen, a single co-pathogen was identified in five [human metapneumovirus (HMPV), parainfluenza virus 2, influenza A virus, rhinovirus, and methicillin-sensitive *Staphylococcus aureus*] and both respiratory syncytial virus and rhinovirus were detected in one Mp-PCR-positive patient.

### Multivariable analyses

In multivariable analyses, Mp-PCR-positive patients were more likely to be younger (18-29 years versus ≥50 years); adjusted odds ratio (aOR): 11.7, 95% CI: 5.1- 26.6, but were clinically indistinct from Mp-PCR-negative patients (*Table 2*). Mp-PCR-positive patients were more likely to have radiographic evidence of lymphadenopathy (aOR: 3.5, 95% CI: 1.2- 9.3) and less likely to have leukocytosis (aOR: 0.4, 95% CI: 0.2- 0.8), rhinorrhea (aOR 0.3, 95% CI: 0.1 - 0.7), or asthma (aOR 0.3, 95% CI: 0.1 - 0.9) (*Table 2*).

## Discussion

In this large multi-center active surveillance study with prospective enrollment and systematic microbiological testing, Mp was detected among approximately 2% of adults hospitalized with CAP and was more prevalent among younger adults aged 18-49 years, which is different from the age distribution for other types of pneumonia 28. Among Mp-PCR-positive patients, illness was not severe, and the median LOS was 2 days. Only four adults with Mp were admitted to the ICU; all had comorbidities. No adult required invasive mechanical ventilation or died. Symptoms and clinical features were not sufficiently distinct in adults to differentiate CAP due to Mp from other etiologies.

This prospective population-based study uniquely uses PCR as the diagnostic test of choice for *M. pneumoniae*. PCR is the current gold standard test for Mp detection due to its higher specificity compared to traditional methods such as serology 32,33. Serology also lacks timeliness due to the need to obtain a convalescent sample to confirm infection. Mycoplasma is fastidious and difficult to grow, so culture is not routinely available; it is also a resource- and labor-intensive method, resulting in this methodology's discontinuation in clinical laboratories 34. Although Food and Drug Administration (FDA)-approved and validated laboratory-developed assays (mostly multiplex assays) are now available for Mp detection, usage remains disappointingly low 32, 35. The role of Mp in adults is likely underestimated clinically, and improving access to PCR testing could lead to opportunities to tailor antibiotic therapy to treat Mp infection, as co-detection of other bacteria was rare.

Macrolide antibiotics, such as azithromycin, are recommended first-line therapy for Mp CAP in adults; other options include fluoroquinolones and tetracyclines 36. Macrolide resistance was only detected in one isolate. During hospitalization, all Mp-PCR-positive patients received antibiotics with activity against Mp. However, the role of antibiotics in the treatment of Mp CAP remains unclear; our study was not designed to address this question and had an inadequate sample size to determine the role of antibiotics in general or, specifically, the role of macrolides in Mp treatment. However, Mp-PCR-positive patients who apparently failed outpatient antibiotic treatment had more commonly received penicillins, suggesting that the diagnosis of mycoplasma by PCR would facilitate appropriate treatment if Mp infection was confirmed.

Our study has several limitations. The study was completed several years ago when the availability of multiplex PCR was less common, but PCR was the standard method for diagnosis in this study. To reduce misclassification, we excluded patients with no detected pathogens from the analyses. It is possible that these patients may have been in their convalescent phase or that available diagnostic testing did not detect Mp 28. The unavailability of all specimen types among the enrolled individuals could have been another factor. Invasive procedures to obtain specimens directly from the lung were not usually performed, and results were only available as part of routine clinical care 28. Another limitation was the difficulty in assessing the role of each pathogen in causing pneumonia when co-detections occurred. No Mp detections were found among a convenience sample of healthy adult controls without respiratory symptoms obtained from an outpatient population (*Supplementary Material*), suggesting that asymptomatic infection is rare. In the EPIC study, patients 65 years or older and those undergoing invasive mechanical ventilation were less likely to be enrolled 28. Also, overall, the enrolled patients in the EPIC study were probably less sick than the non-enrolled patients based on the exclusion criteria. Finally, our findings may not be representative of other settings because all study sites were located in urban U.S. medical centers.

## Conclusions

*Mycoplasma pneumoniae* was the second most common bacteria detected among adults hospitalized with CAP, with a higher prevalence among younger adults 28. The illness was not severe, and clinical signs and symptoms were not distinct between Mp and other respiratory pathogens. Improved access to and validation of point-of-care diagnostic testing using PCR for Mp detection may facilitate prompt and appropriate clinical triage and antibiotic therapy, particularly in younger adults who present with CAP.

## Supplementary Material

Supplementary information and tables.

## Figures and Tables

**Table 1:** Epidemiologic features among adults hospitalized for CAP with and without M. pneumoniae detection*, Etiology of Pneumonia in the Community (EPIC) study, January 2010—June 2012 (n=853)

Characteristic	*M. pneumoniae* PCR-positive* (n=43) N (%)	*M. pneumoniae* PCR-negative with other detected pathogens* (n=810) N (%)	Unadjusted Odds Ratio (95% CI)	P-value
*Demographics* Age in years 18-29 30-49 ≥50	14 (33) 12 (28) 17 (39)	57 (7) 194 (24) 559 (69)	8.1 (3.8 - 17.2) 2.0 (1.0 - 4.3) *Reference*	<0.01 0.3
Male	22 (51)	378 (47)	1.2 (0.6 - 2.2)	0.6
Race/ethnicity^†^				
Hispanic	8 (19)	95 (12)	1.4 (0.6 - 3.1)	0.5
Non-Hispanic Black	11 (25)	301 (37)	0.6 (0.3 - 1.2)	0.1
Non-Hispanic White	24 (56)	385 (48)	*Reference*	
***Clinical Presentation***				
Duration of symptoms prior to admission				
Median (IQR) in days	6.6 (3.7- 8.8)	4.1 (2.4- 7.5)		<0.01^a^
Cough	43 (100)	735 (91)	NC	0.03^f^
Fever/feverish	37 (86)	593 (73)	2.3 (0.9 - 5.4)	0.06
Fatigue	36 (84)	645 (80)	1.3 (0.6 - 3.0)	0.5
Chills	33 (77)	559 (69)	1.5 (0.7 - 3.1)	0.3
Dyspnea	27 (63)	630 (78)	0.5 (0.3 - 0.9)	0.02
Chest pain	27 (63)	375 (46)	2.2 (1.03-3.7)	0.03
Headache	27 (63)	414 (51)	1.6 (0.9 - 3.0)	0.1
Myalgia	25 (58)	370 (46)	1.7 (0.9- 3.1)	0.1
Wheezing	21 (49)	382 (47)	1.1 (0.6 - 2.0)	0.8
Nausea	21 (49)	310 (38)	1.5 (0.8 - 2.8)	0.2
Sore throat	12 (28)	286 (35)	0.7 (0.4 - 1.4)	0.3
Diarrhea	11 (26)	192 (24)	1.1 (0.5 - 2.2)	0.8
Rhinorrhea	9 (21)	364 (45)	0.3 (0.2 - 0.7)	<0.01
***Medical history***				
Any comorbid condition	33 (77)	734 (91)	0.3 (0.2 - 0.7)	0.01
Obesity^b^	28 (67)	516 (64)	1.1 (0.6 - 2.1)	0.8
Coronary artery disease	11 (26)	229 (28)	0.9 (0.4 - 1.8)	0.7
Diabetes mellitus	5 (12)	216 (27)	0.4 (0.1 - 0.9)	0.03
Asthma	4 (9)	209 (26)	0.3 (0.1 - 0.8)	0.01
Chronic obstructive pulmonary disease	3 (7)	186 (23)	0.3 (0.1-0.8)	0.01
Renal disorder	2 (5)	120 (15)	0.3 (0.1-1.2)	0.06
Cancer	2 (5)	165 (20)	0.2 (0.1-0.8)	0.01
Smoker (current)	9 (21)	237 (29)	0.6 (0.3 - 1.4)	0.2
***Exam findings***				
Rales	22 (51)	340 (42)	1.4 (0.8 - 2.7)	0.2
Fever	17 (40)	248 (31)	1.5 (0.8 - 2.8)	0.2
Tachypnea^c^	16 (37)	322 (40)	0.9 (0.5 - 1.7)	0.7
Wheeze	11 (26)	291 (36)	0.6 (0.3 - 1.2)	0.2
Decreased breath sounds	11 (26)	240 (30)	0.8 (0.4 - 1.6)	0.6
Rhonchi	9 (21)	236 (29)	0.6 (0.3 - 1.4)	0.2
Hypoxia^d^	5 (12)	166 (20)	0.5 (0.2 - 1.3)	0.2
***Radiologic findings^e^***				
Consolidation	34 (79)	517 (64)	2.1 (1.01 - 4.5)	0.04
Single lobar infiltrate	18 (42)	259 (32)	1.5 (0.8 - 2.9)	0.2
Multiple lobar infiltrate	16 (37)	233 (29)	1.5 (0.8 - 2.8)	0.2
Air space/ interstitial diseases	13 (30)	307 (38)	0.7 (0.4 - 1.4)	0.3
Pleural effusion	9 (21)	212 (26)	0.7 (0.4 - 1.6)	0.4
Hilar lymphadenopathy	6 (14)	48 (6)	2.6 (1.03 - 6.4)	0.05^f^
***Laboratory findings***				
Hyponatremia^g^	19 (45)	264 (33)	1.7 (0.9 - 3.1)	0.1
Leukocytosis^h^	12 (28)	400 (49)	0.4 (0.2- 0.8)	<0.01
***Severity***				
Length of hospital stay (median, IQR in days)	2 (1-4)	4 (2-6)		<0.01
PSI Class I^i^	23 (54)	151 (19)	*Reference*	
PSI Class II^i^	10 (23)	211 (26)	0.2 (0.07 -0.3)	<0.01
PSI Class III-V^i^	10 (23)	448 (55)	0.3 (0.1 - 0.7)	<0.01
ICU admission	4 (9)	210 (26)	0.3 (0.1- 0.8)	0.01
Mechanical ventilation	0	62 (29)	NC	0.08^f^
Death	0	16 (2)	NC	0.7^f^
***Antibiotics***				
Receipt of an outpatient antibiotic	10 (23)	133 (16)	1.5 (0.7 - 3.2)	0.2
Receipt of antibiotics within 5 days prior to admission^j^	9 (21)	86 (11)	2.2 (1.03 - 4.8)	0.04^f^
*Penicillins*^k^	5 (56)	15 (17)	*Reference*	
*Macrolides*	1 (11)	34 (40)	0.1 (0.01 - 0.8)	0.02^f^
*Cephalosporin*	1 (11)	3 (3)	1.0 (0.08- 11.9)	1.0^ f^
*Quinolones*	1 (11)	21 (24)	0.1 (0.02 - 1.4)	0.1^ f^

Abbreviations: CI, Confidence interval; NC: Could not be calculated as one cell contains a zero Note: Etiology of Pneumonia in the Community (EPIC) study, 2010-2012 *M. pneumoniae PCR-positive: A radiographically confirmed CAP patient enrolled in EPIC with a positive *M. pneumoniae* PCR. *M. pneumoniae* PCR-negative with another pathogen detected: A patient enrolled in EPIC with radiographically confirmed pneumonia who had a negative M. pneumoniae PCR result but had another pathogen detected, such as a bacterial or viral pathogen. ^†^The race/ethnicity of the 29 Mp-PCR negative not presented are Non-Hispanic Asian (n=15), Non-Hispanic Hawaiian/Pacific Islander (n=1), Non-Hispanic American Indian/American Native (n=6), multiracial (n=3), and other (n=4) ^a^ Wilcoxon Two-sample test ^b^ Body mass index (BMI) was calculated as weight (kg)/height (m)2; categories included underweight (<18.5 kg/m2), normal weight (18.5-24.9 kg/m2), and obese (≥ 25 kg/m2). BMI was missing for one Mp-PCR-positive and seven Mp-PCR-negative patients ^c^Tachypnea: >20 breaths/min were considered as abnormal ^d^Hypoxia: Oxygen saturation rate (SpO2) <90% on admission using pulse oximetry on room air or a fraction of inspired oxygen (FiO2) of >0 L or >21% at presentation ^e^ The radiographic findings are not mutually exclusive and could overlap ^f^ Fisher's exact ^g^ Serum sodium <135 U/L. For Mp-PCR-positive the denominator is 42 and for Mp-PCR-negative the denominator is 799 ^h^ WBC >11,000/mm^3^ was considered abnormal. For Mp-PCR-negative, the denominator is 797 ^i^The categories were Class 1 (PSI score 0-50 points), Class II (PSI score 51-70 points), Class III (PSI 71 - 90 points), Class, IV (PSI score 91 - 130 points), and Class V (PSI score 131 - 395 points). ^j^The percentages are based on those who received an antibiotic within 5 days prior to admission ^k^Penicillins included amoxicillin, penicillin, ampicillin-sulbactam, amoxicillin, and amoxicillin-clavulanate

**Table 2:** Multivariable predictors* for M. pneumoniae detection among U.S. adults (≥18 years) hospitalized for CAP compared to persons with CAP due to other pathogens, Etiology of Pneumonia in the Community (EPIC) study, January 2010—June 2012

Characteristics		Adjusted OR (95% CI)	Adjusted P value
Age in years	18-29	11.7 (5.1 - 26.6)	<0.01
	30-49	2.0 (0.9 - 4.4)	0.2
	≥50	*Reference group*	
Hilar lymphadenopathy		3.4 (1.2 - 9.3)	0.02
Receipt of antibiotics 5 days before admission		2.4 (1.01 - 5.5)	0.045
Leukocytosis		0.4 (0.2 - 0.8)	0.01
Rhinorrhea		0.3 (0.2 - 0.7)	<0.01
History of Asthma		0.3 (0.1 - 0.9)	0.02

Abbreviations: CI, Confidence interval Note: Etiology of Pneumonia in the Community (EPIC) study, 2010-2012 *Variables that were tested in the model but did not reach significance: hospital site, sex; clinical presentation of fever/feverish, dyspnea, chest pain; leukocytosis, radiographic findings: consolidation; history of chronic obstructive pulmonary disease, renal disease, cancer; receipt of antibiotics within 5 days prior to admission; duration of symptoms prior to admission; household size; interaction terms: age and renal disease, age and diabetes mellitus, age and cancer, hospital site and receipt of antibiotics 5 days prior to admission
