# Supplementary material for: Clinical and Epidemiologic Features of Mycoplasma pneumoniae Infection Among Adults Hospitalized with Community-acquired Pneumonia
Source: Int J Med Sci. 2024 Nov 11;21(15):3003–9. doi: 10.7150/ijms.99233 (PMC11610339; doi:10.7150/ijms.99233)
Supplement: Supplementary file 1 — Supplementary information and tables. [file ijmsv21p3003s1.pdf]

Supplementary Material

Statistical analysis – additional definitions

Viral pneumonia was defined as pneumonia with adenovirus (AdV); coronaviruses; human metapneumovirus (hHMV); human rhinovirus (HRV); influenza A/B viruses; parainfluenza viruses (PIV 1, 2, 3); respiratory syncytial virus (RSV); and Coronavirus (CoV). Bacterial pneumonia was defined as pneumonia with bacterial detection by the methods described in the laboratory methods (below). Patients in this category could have had more than one bacterium detected, but no atypical bacteria (Legionella or *C. pneumoniae*) or virus. Fungal infections were not included. If *M. pneumoniae* (Mp) was not detected by PCR and no other pathogen was detected, the patient was considered to have CAP without Mp (Mp-PCR-negative without a pathogen detected).

A white blood count (WBC) >11,000/mm^3 and serum sodium <135 U/L were considered abnormal. Body mass index (BMI) was calculated as weight (kg)/height (m)^2; categories included underweight (<18.5 kg/m^2), normal weight (18.5-24.9 kg/m^2), and obese (≥ 25 kg/m^2). Antibiotics considered to be active against Mp included macrolides (e.g., erythromycin, azithromycin, and clarithromycin), fluoroquinolones (e.g., levofloxacin and moxifloxacin), and doxycycline. An outpatient antibiotic exposure was defined as self-reported antibiotic use within five calendar days prior to hospitalization; information on the day but not hour was collected for outpatient antibiotic exposures. Inpatient antibiotic exposure was defined as receipt of an antibiotic at any time after admission.

Laboratory methods

Briefly, Gram staining and bacterial culture were performed on blood and lower respiratory (including pleural fluid) samples at each study site using standard techniques [1]. Using PCR, pleural fluid was also tested at the University of Utah for bacterial pathogens (*H. influenzae* and other Gram-negative bacteria, *Staphylococcus aureus*, *Streptococcus anginosus/mitis*, *S. pneumoniae*, and *S.*

pyogenes). At each study site, nasopharyngeal/oropharyngeal (NP/OP) swabs were tested using CDC-developed real-time PCR methods for detection of viruses, including adenovirus (AdV), coronaviruses (CoV); human metapneumovirus (HMV); human rhinovirus; influenza A/B viruses; parainfluenza viruses (PIV 1,2,3); and respiratory syncytial virus (RSV) and bacteria including *Chlamydophila pneumoniae*; in addition to *M. pneumoniae*. Quality assurance and monitoring protocols maintained standardization among sites. Serology for certain viruses such as AdV, HMPV, influenza A/B, PIV, and RSV was performed at CDC on available paired acute and convalescent sera. Microbiologic results from tests conducted for clinical care, including fungi or mycobacterium, were also obtained and included.

Macrolide susceptibilities:

Post-amplification high-resolution melt (HRM) analysis allows the detection of an A to G transition at position 2063 or 2064 within the 23S rRNA gene, the two mutations most commonly associated with macrolide resistance in Mp[1]. Melt profiles are classified as sensitive or resistant by comparison to reference strains included in each run. Sequencing analysis was performed on all isolates classified as resistant based on the melt profile to identify the specific single-base mutation (A2063G or A2064G) in the 23S rRNA gene. The details of this methodology have been described elsewhere[1,2].

Of the 43 Mp PCR-positive specimens, 40 (93%) were positive upon repeat PCR testing at CDC. Of these, 33 (83%) Mp isolates were recovered, and 1 (3%) was macrolide-resistant. The one patient with macrolide resistance was a 28-year-old male with a single lobar consolidation on chest radiography who was admitted to the general ward for 2 days and discharged. There was no documented receipt of

[1] Wolff BJ, Thacker WL, Schwartz SB, Winchell JM. Detection of macrolide resistance in *Mycoplasma pneumoniae* by real-time PCR and high-resolution melt analysis. Antimicrob Agents Chemother **2008**; 52(10): 3542-9.

[2] Diaz MH, Benitez AJ, Cross KE, et al. Molecular Detection and Characterization of Mycoplasma pneumoniae Among Patients Hospitalized With Community-Acquired Pneumonia in the United States. Open forum infectious diseases **2015**; 2(3): ofv106.

an antibiotic with activity against Mp before admission. Upon hospitalization, he received azithromycin (1 day) and ceftriaxone (1 day) and was discharged on levofloxacin.

Several published reports of macrolide-resistant Mp in the United States and internationally have raised public health concerns about the potential for macrolide treatment failures. However, the clinical implications are still unclear.[2,3,4]

Bivariate analysis: Comparison with Mp-PCR-negative without a detected pathogen

In unadjusted analyses, Mp-PCR-positive patients were more likely to be younger than Mp-PCR-negative patients without a pathogen detected (*Supplemental Table 3*). Mp-PCR-positive patients had a longer duration of symptoms before hospitalization (median: 6.6 vs. 4.3 days; P<0.01) and were more likely to report cough (100% vs. 85%; P<0.01), fever (86% vs. 65%, P<0.01), headache (63% vs 44%, P=0.01), nausea (49% vs 33%, P=0.03), or have hyponatremia (45% vs 24%, P<0.01) or radiographic consolidation (79% vs. 61%, P = 0.02) but less likely to report dyspnea (63% vs. 79%, P=0.01) or comorbidities (77% vs. 92%, P<0.01) or have leukocytosis (28% vs. 55%, P <0.01) compared with Mp-PCR-negative patients without a pathogen detected (*Supplemental Table 3*). Other than fever, no significant clinical exam findings were identified. Among Mp-PCR-positive patients, there were 4 (9%) ICU admissions when compared to 267 (19%) ICU admissions among Mp-PCR-negative patients without a pathogen detected. No death was reported among Mp-PCR-positive patients during hospitalization, while 33 (2%) of Mp-PCR-negative without a pathogen detected patients died. Overall, median hospital LOS was shorter (2 vs. 3 days, P=0.05) for Mp-PCR-positive versus Mp-PCR-negative patients without a pathogen detected.

---

[3] Miyashita N, Akaike H, Teranishi H, Ouchi K, Okimoto N. Macrolide-resistant *Mycoplasma pneumoniae* pneumonia in adolescents and adults: clinical findings, drug susceptibility, and therapeutic efficacy. Antimicrob Agents Chemother **2013**; 57(10): 5181-5.

[4] Watkins LKF, Olson D, Diaz MH, et al. Epidemiology and molecular characteristics of *Mycoplasma pneumoniae* during an outbreak of *M. pneumoniae*-associated Stevens-Johnson Syndrome. Pediatr Infect Dis J **2017**; 36(6): 564-71

Control enrollment

A convenience sample of asymptomatic adults from the Nashville study site (n=238) who presented for non-acute care to a general medicine clinic were enrolled weekly from November 1, 2011, to June 30, 2012 [1]. NP/OP swabs were obtained to assess the prevalence of respiratory pathogens. Exclusion criteria for the adults with pneumonia were also applicable to control adults. In addition, controls were excluded if they had fever or respiratory symptoms within 14 days before or after enrollment or had received live attenuated influenza vaccination within 7 days before enrollment. Mp was not detected among a convenience sample of asymptomatic adult controls.

Supplemental Table 1 (S1): ***Demographics of adults hospitalized for M. pneumoniae community-acquired pneumonia*, according to the site, Etiology of Pneumonia in the Community (EPIC) study, January 2010—June 2012*** **(n=43)**

Characteristics	***Chicago*** n=33 n%	***Nashville*** n=10 n%	***Overall*** (n=43) n (%)
Proportion of *M. pneumoniae* CAP **	33/1516 (2%)	10/756 (1%)	43/2272 (2%)
**Age (*years*)**			
Median (IQR)	45 (29 – 53)	41.5 (28 – 50)	45 (28 – 53)
**Age groups**			
18-29 years	11 (33)	3 (30)	14 (33)
30-49 years	8 (24)	4 (40)	12 (28)
≥ 50 years	14 (42)	3 (30)	17 (39)
**Sex**			
Male	17 (52)	5 (50)	22(60)
Female	16 (48)	5 (50)	21 (40)
**Race/Ethnicity**			

Non-Hispanic white	18 (56)	6 (60)	24 (56)
Non-Hispanic black	7 (21)	4 (40)	11 (25)
Hispanic	8 (24)	0	8 (19)
**Insurance**			
Public	12 (36)	3 (30)	15 (35)
Private	11 (33)	4 (40)	15 (35)
Both	0	1 (10)	1 (2)
None	9 (27)	2 (20)	11 (26)

Note: Etiology of Pneumonia in the Community (EPIC) study, 2010-2012
**M. pneumoniae* PCR-positive CAP: A radiographically confirmed CAP patient enrolled in EPIC with a positive *M. pneumoniae* PCR.
** Proportion of all Nasopharyngeal/oropharyngeal specimens that tested positive for *M. pneumoniae*
Note: Percentages may not add up to 100 because of rounding

Supplemental Table 2 (S2): ***Select epidemiologic features among adults hospitalized for CAP with M. pneumoniae and those without M. pneumoniae *, Etiology of Pneumonia in the Community (EPIC) study, January 2010—June 2012*** **(n=3024)**

Characteristic	*M. pneumoniae* PCR-positive*	Bacterial pneumonia (excludes atypical)*		Viral pneumonia*		*M. pneumoniae* PCR-negative with no pathogen detected*	
	(n=43)	(n=169)	Unadjusted Odds Ratio (95% CI)	(n=583)	Unadjusted Odds Ratio (95% CI)	(n=1419)	Unadjusted Odds Ratio (95% CI)
	n%	n%		n%		n%	
Demographics							
Age in years							
18-29	14 (33)	8 (5)	13.6 (5.0 - 37.1) †	47 (8)	6.9 (3.2 - 14.8) †a	81 (6)	10.3 (4.9 – 21.6) †
30-49	12 (28)	29 (17)	3.2 (1.4- 7.5) ‡	145 (25)	1.9 (0.9 - 4.1)	328 (23)	2.2 (1.0- 4.6)
≥50	17 (39)	132 (78)	*Reference*	391 (67)	*Reference*	1010 (71)	*Reference*
Male	22 (51)	85 (50)	1.0 (0.5 - 2.0)	251 (43)	1.4 (0.7 - 2.6)	708 (50)	1.1 (0.6 – 1.9)
Race/ethnicity							
Hispanic	8 (18)	14 (8)	2.3 (0.9 - 6.1)	70 (12)	1.3 (0.5 - 2.9)	136 (10)	1.6 (0.7 – 3.6)
Non-Hispanic Black	11 (26)	54 (32)	0.8 (0.4 - 1.8)	225 (39)	0.5 (0.3 - 1.1)	568 (40)	0.5 (0.3- 1.1)
Non-Hispanic White	24 (56)	97 (57)	*Reference*	266 (46)	*Reference*	652 (46)	*Reference*
Clinical Presentation							
Duration of symptoms prior to admission in days [Median (IQR) in days]	6.6 (3.7-8.8)	3.7 (1.7 - 7.5) †b		4.0 (2.4 - 7.4) †b		4.3 (1.9 -9.0) †b	
Cough	43 (100)	134 (79)	NC†a	551 (95)	NCa	1211 (85)	NC†a
With sputum	29 (67)	79 (47)	2.4 (1.2 - 4.8) §	385 (66)	1.1 (0.6 - 2.1)	733 (52)	2.0 (1.0 – 3.7) §

With blood	3 (7)	19 (11)	0.6 (0.2 - 2.1) [a]	41 (7)	1.0 (0.3 - 3.3)	126 (9)	0.8 (0.2 – 2.5) [a]
Fever/feverish	37 (86)	112 (66)	3.1 (1.3 - 7.9) ‡	431 (74)	2.2 (0.9 - 5.3)	91 (65)	3.5 (1.4 – 8.1) †
Fatigue	36 (84)	135 (80)	1.3 (0.5 - 3.2)	464 (80)	1.3 (0.6 - 3.0)	1131 (80)	1.3 (0.6 - 3.0)
Chills	33 (77)	117 (69)	1.5 (0.7 - 3.2)	405 (69)	1.5 (0.7 - 3.0)	903 (64)	1.9 (0.9 - 3.9)
Dyspnea	27 (63)	126 (75)	0.6 (0.3 - 1.2)	467 (80)	0.4 (0.2 - 0.8) †	1120 (79)	0.5 (0.2 – 0.8) ‡
Appetite	27 (63)	93 (55)	1.4 (0.7 - 2.7)	318 (55)	1.4 (0.7 - 2.7)	725 (51)	1.6 (0.9 - 3.0)
Chest pain	27 (63)	77 (46)	2.0 (1.01 - 4.0) §	278 (48)	1.9 (1.0 - 3.5)	716 (50)	1.7 (0.9 - 3.1)
Headache	27 (63)	72 (43)	2.3 (1.1 - 4.5) §	309 (53)	1.5 (0.8 - 2.8)	617 (44)	2.2 (1.2 – 4.1) ‡
Myalgia	25 (58)	79 (47)	1.6 (0.8 - 3.1)	261 (45)	1.7 (0.9 - 3.2)	623 (44)	1.8 (1.0 3.3)
Wheezing	21 (49)	66 (39)	1.5 (0.8 - 2.9)	307 (53)	0.9 (0.5 - 1.6)	586 (41)	1.4 (0.7 - 2.5)
Nausea	21 (49)	67 (40)	1.5 (0.7 - 2.8)	223 (38)	1.5 (0.8 - 2.9)	471 (33)	1.9 (1.1 – 3.5) §
Sore throat	12 (28)	45 (27)	1.1 (0.5 -2.3)	229 (39)	0.6 (0.3 - 1.2)	360 (25)	1.1 (0.6 - 2.2)
Diarrhea	11 (26)	40 (24)	1.1 (0.5 - 2.4)	133 (23)	1.2 (0.6 - 2.4)	281 (20)	1.4 (0.7 - 2.8)
Rhinorrhea	9 (21)	62 (37)	0.5 (0.2 - 1.0)	291 (50)	0.3 (0.1 - 0.6) †	484 (34)	0.5 (0.2 – 1.1)
Abdominal pain	9 (21)	38 (22)	0.9 (0.4 - 2.1)	130 (22)	0.9 (0.4 - 2.0)	289 (20)	1.0 (0.5 - 2.2)
Confusion	9 (21)	42 (25)	0.8 (0.4 - 1.8)	42 (25)	0.8 (0.4 - 1.8)	269 (19)	1.1 (0.5 - 2.4)
Medical history							
Any comorbid condition	33 (77)	156 (92)	0.3 (0.1 - 0.7) ‡[a]	529 (91)	0.3 (0.2 - 0.7) †[a]	1131 (80)	0.2 (0.1-0.3) †
Obesity[c]	28 (67)	100(59)	0,7 (0.4 - 1.5)	378 (65)	1.0 (0.5 - 1.8)	896 (64)	1.1 (0.6 - 2.2)
Coronary Artery Disease	11 (26)	54 (32)	0.7 (0.3 - 1.6)	194 (33)	0.8 (0.4 - 1.5)	424 (30)	0.8 (0.4 - 1.6)
Cardiac Failure	6 (14)	43 (25)	0.5 (0.2 - 1.2)	106 (18)	0.7 (0.3 - 1.8)	269 (19)	0.7 (0.3 - 1.7)
Diabetes mellitus	5 (12)	54 (32)	0.3 (0.1 - 0.8) †	145 (25)	0.4 (0.2 - 1.0)	362 (26)	0.4 (0.2 – 0.98) §
Asthma	4 (9)	28 (17)	0.5 (0.2 - 1.6)	170 (29)	0.2 (0.9 - 0.7) †	375 (26)	0.3 (0.1 – 0.8) ‡
Chronic Obstructive Pulmonary Disease (COPD)	3 (7)	50 (30)	0.2 (0.05 - 0.6) †	133 (23)	0.3 (0.1 - 0.8) §	334 (24)	0.2 (0.08-0.8) ‡
Renal disorder	2 (5)	39 (23)	0.2 (0.04 - 0.7) †	73 (13)	0.3 (0.1 - 1.4)	237 (17)	0.2 (0.06 -1.01) §
Cancer	2 (5)	44 (26)	0.1 (0.03 - 0.6) †	116 (20)	0.2 (0.05 - 0.8) ‡	294 (21)	0.2 (0.05-0.8) ‡
Smoker (current)	9 (21)	47 (28)	0.7 (0.3 - 1.5)	177 (30)	0.6 (0.3 - 1.3)	350 (25)	0.8 (0.4 - 1.7)

Exam findings							
Rales	22 (51)	67 (40)	1.6 (0.8 - 3.1)	242 (42)	1.5 (0.8 - 2.7)	647 (46)	1.3 (0.7 – 2.3)
Fever	17 (40)	51 (30)	1.5 (0.8 - 3.0)	174 (30)	1.5 (0.8 - 2.9)	316 (22)	2.3 (1.2 – 4.3) †
Tachypnea[d]	16 (37)	71 (42)	0.8 (0.4 - 1.6)	234 (40)	0.9 (0.5 - 1.7)	517 (36)	1.0 (0.6 - 1.9)
Wheeze	11 (26)	45 (27)	0.9 (0.4 - 2.0)	240 (41)	0.5 (0.2 - 0.9)§	347 (25)	1.1 (0.5 - 2.1)
Decreased breath sounds	11 (26)	55 (33)	0.7 (0.3 - 1.5)	170 (29)	0.8 (0.4 - 1.7)	451 (32)	0.7 (0.4 - 1.5)
Rhonchi	9 (21)	51 (30)	0.6 (0.3 - 1.4)	175 (30)	0.6 (0.3 - 1.3)	302 (21)	1.0 (0.5 - 2.1)
Hypoxia[e]	5 (12)	49 (29)	0.3 (0.1 - 0.9) §	113 (19)	0.5 (0.2 - 1.4)	287 (20)	0.5 (0.2 - 1.3)
Dullness to percussion	2 (5)	5 (3)	1.6 (0.3 - 8.5) [a]	7 (1)	4.0 (0.8 - 19.9)[a]	31 (2)	2.2 (0.5 - 9.4)
Radiologic findings[f]							
Consolidation	34 (79)	114 (67)	1.8 (0.8 - 4.1)	351 (60)	2.5 (1.2 - 5.3)‡	863 (61)	2.4 (1.2 – 5..1) §
Single lobar infiltrate	18 (42)	50 (30)	1.7 (0.9 - 3.4)	176 (30)	1.7 (0.9 - 3.1)	425 (30)	1.7 (0.9 – 3.1)
Multiple lobar infiltrate	16 (37)	52 (31)	1.3 (0.7 - 2.7)	164 (28)	1.5 (0.8 - 2.9)	413 (29)	1.4 (0.8 - 2.7)
Air space/ interstitial diseases	13 (30)	59 (35)	0.8 (0.4 - 1.7)	237 (41)	0.6 (0.3 - 1.2)	587 (41)	0.6 (0.3 - 1.2)
Pleural effusion	9 (21)	62 (37)	0.5 (0.2 - 1.0)	135 (23)	0.9 (0.4 - 1.9)	473 (33)	0.5 (0.3 – 1.1)
Hilar lymphadenopathy	6 (14)	11 (7)	2.3 (0.8 - 6.7) [a]	27 (5)	3.3 (1.3 - 8.6) §[a]	102 (7)	2.1 (0.9 - 5.1) [a]
Laboratory findings							
Hyponatremia	19 (45)	63 (37)	1.4 (0.7 - 2.8)	167 (29)	2.0 (1.1 - 3.8)§	343 (24)	2.6 (1.4 – 4.8) †
Leukocytosis[h]	12 (28)	110 (65)	0.2 (0.1 - 0.4) †	261 (46)	0.5 (0.2 - 0.9)§	766 (55)	0.3 (0.2 – 0.6) †
Severity							
Length of stay (median, IQR, days)	2 (1-4)	6 (3- 11) †[b]		3 (2- 5)§[b]		3 (2-6) †	
ICU admission	4 (9)	79 (47)	0.1 (0.04 - 0.3) †	122 (21)	0.4 (0.1 - 1.1)	267 (19)	0.4 (0.2 - 1.2)
Ventilation (subset of ICU)	0	30 (38)	NC[a]	28 (23)	NC[a]	59 (22)	NC[a]

PSI Class I[i]	23 (54)	20 (12)	*Reference*	115 (20)	*Reference*	248 (17)	*Reference*
PSI Class II[i]	10 (23)	28 (17)	0.3 (0.1 - 0.8) §	166 (28)	0.2 (0.08 - 0.4) †	387 (27)	0.1 (0.07 - 0.3) †
PSI Class III-V[i]	10 (23)	121 (72)	0.07 (0.03 - 0.2) †	302 (52)	0.3 (0.1 - 0.7) †	784 (55)	0.3 (0.1 - 0.6) †
Antibiotics							
Receipt of an outpatient antibiotic	10 (23)	18 (11)	2.5 (1.1- 6.0) §	104 (18)	1.4 (0.7 - 2.9)	302 (21)	1.1 (0.5-2.3)
Receipt of antibiotics within 5 days prior to admission[j]	9 (21)	15	2.7 (1.1- 6.7) §a	63 (11)	2.2 (1.0 - 4.8)	145 (10)	2.3 (1.1 – 4.9) §a
Penicillins[k]	5 (56)	3	*Reference*	9 (14)	*Reference*	27 (19)	*Reference*
Macrolides	1 (11)	5	0.1 (0.01 - 1.6) a	27 (43)	0.1 (0.01 - 0.6) §a	57 (39)	0.1 (0.01 - 0.9) §a
Cephalosporin	1 (11)	1	0.6 (0.03 -13.6) a	3 (5)	0.6 (0.05 - 7.4) a	4 (3)	1.4 (0.1 - 14.7) a
Quinolones	1 (11)	5	0.1 (0.01 - 1.6) a	14 (22)	0.1 (0.01 - 1.3) a	56 (39)	0.1 (0.01 - 0.9) §a

Abbreviations: CI, Confidence interval; NC: Could not be calculated as one cell contains a zero
Note: Etiology of Pneumonia in the Community (EPIC) study, 2010-2012
**M. pneumoniae* PCR-positive CAP: A radiographically confirmed CAP patient enrolled in EPIC with a positive *M. pneumoniae* PCR.
Viral pneumonia was defined as pneumonia with adenovirus (AdV); coronaviruses; human metapneumovirus (hHMV); human rhinovirus (HRV); influenza A/B viruses; parainfluenza viruses (PIV 1,2,3); respiratory syncytial virus (RSV); and Coronavirus (CoV).
Bacterial pneumonia was defined as pneumonia with either bacterial detection by the methods described in the Laboratory methods. Patients in this category could have had more than one bacteria detected, but no atypical bacteria (*Legionella* or *Chlamydophila pneumoniae*) or virus.
Fungal infections were not included.
If *M. pneumoniae* was not detected by PCR and no other pathogen was detected, the patient was considered to have CAP without *M. pneumoniae* (Mp-PCR-negative without a pathogen detected).
†P<0.01
‡P=0.01
§P<0.05
[a] Fishers
[b] Wilcoxon Two-sample test
[c] Body mass index (BMI) was calculated as weight (kg)/height (m)2; categories included underweight (<18.5 kg/m2), normal weight (18.5-24.9 kg/m2), and obese (≥ 25 kg/m2).
[d]Tachypnea: >20 breaths/min were considered as abnormal
[e]Hypoxia: Oxygen saturation rate (SpO2) <90% on admission using pulse oximetry on room air or a fraction of inspired oxygen (FiO2) of >0 L or >21% at presentation
[f]The radiographic findings are not mutually exclusive and could overlap
[g] Serum sodium <135 U/L . For Mp-PCR-positive the denominator is 42 and for Mp-PCR-negative the denominator is 799
[h] WBC >11,000/mm^3 was considered abnormal. For Mp-PCR-negative the denominator is 797
[i]The categories were Class 1 (PSI score 0–50 points), Class II (PSI score 51–70 points), Class III (PSI 71 – 90 points), Class, IV (PSI score 91 – 130 points), and Class V (PSI score 131 – 395 points).

[j]The percentages are based on those who received an antibiotic within 5 days prior to admission
